# 4-Nitro­phenyl­hydrazinium picrate monohydrate

**DOI:** 10.1107/S1600536813032479

**Published:** 2013-12-07

**Authors:** Hong-lan Cai, Bing Liu, Qing-an Qiao

**Affiliations:** a School of Chemistry and Material Science, Ludong University, Yantai 264025, People’s Republic of China

## Abstract

In the crystal structure of the title compound, C_6_H_8_N_3_O_2_^+^·C_6_H_2_N_3_O_7_^−^·H_2_O, N—H⋯O and O—H⋯O hydrogen bonds link the components into a two-dimensional network parallel to (010). In addition, there are pairs of weak inversion-related C—H⋯O hydrogen bonds within the two-dimensional network. The three nitro groups are twisted by 1.6 (3), 7.8 (3) and 12.1 (3)° from the ring plane in the anion, while in the cation, the nitro group makes a dihedral angle of 4.6 (2)° with the ring.

## Related literature   

For the use of picric acid acid as a co-crystallization agent, see: Herbstein & Kaftory (1976 ▶); Dubost *et al.* (1981 ▶); Harrison *et al.* (2007 ▶); Peng *et al.* (2011 ▶); Zeng *et al.* (2011 ▶); Dey *et al.* (2011 ▶).
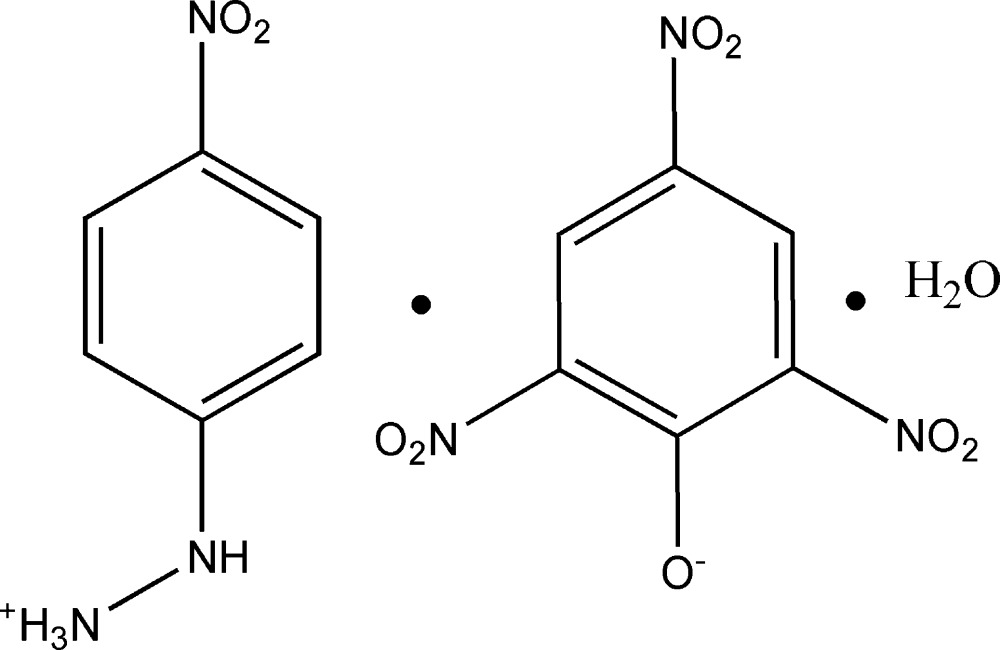


## Experimental   

### 

#### Crystal data  

C_6_H_8_N_3_O_2_^+^·C_6_H_2_N_3_O_7_^−^·H_2_O
*M* *_r_* = 400.28
Monoclinic, 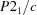

*a* = 4.8483 (3) Å
*b* = 28.798 (2) Å
*c* = 11.6352 (8) Å
β = 101.360 (1)°
*V* = 1592.70 (18) Å^3^
*Z* = 4
Mo *K*α radiation
μ = 0.15 mm^−1^
*T* = 299 K
0.20 × 0.08 × 0.04 mm

#### Data collection  

Bruker SMART CCD area-detector diffractometer
Absorption correction: multi-scan (*SADABS*; Sheldrick, 1997 ▶) *T*_min_ = 0.971, *T*_max_ = 0.994
16632 measured reflections
3643 independent reflections
2487 reflections with *I* > 2σ(*I*)
*R*_int_ = 0.045

#### Refinement  

*R*[*F* ^2^ > 2σ(*F* ^2^)] = 0.063
*wR*(*F* ^2^) = 0.150
*S* = 1.05
3643 reflections
271 parameters
10 restraints
H atoms treated by a mixture of independent and constrained refinement
Δρ_max_ = 0.31 e Å^−3^
Δρ_min_ = −0.31 e Å^−3^

### 

Data collection: *SMART* (Bruker, 2001 ▶); cell refinement: *SAINT-Plus* (Bruker, 2001 ▶); data reduction: *SAINT-Plus*; program(s) used to solve structure: *SHELXS97* (Sheldrick, 2008 ▶); program(s) used to refine structure: *SHELXL97* (Sheldrick, 2008 ▶); molecular graphics: *PLATON* (Spek, 2009 ▶); software used to prepare material for publication: *SHELXTL* (Sheldrick, 2008 ▶).

## Supplementary Material

Crystal structure: contains datablock(s) global, I. DOI: 10.1107/S1600536813032479/lh5673sup1.cif

Structure factors: contains datablock(s) I. DOI: 10.1107/S1600536813032479/lh5673Isup2.hkl

Supporting information file. DOI: 10.1107/S1600536813032479/lh5673Isup3.cml

Additional supporting information:  crystallographic information; 3D view; checkCIF report

## Figures and Tables

**Table 1:** Hydrogen-bond geometry (Å, °)

*D*—H⋯*A*	*D*—H	H⋯*A*	*D*⋯*A*	*D*—H⋯*A*
N1—H1*A*⋯O10^i^	0.87 (1)	2.05 (1)	2.916 (3)	177 (2)
N1—H1*B*⋯O10	0.87 (1)	1.94 (1)	2.809 (3)	172 (2)
N1—H1*B*⋯O9^ii^	0.87 (1)	2.56 (3)	2.908 (3)	105 (2)
N1—H1*C*⋯O6^iii^	0.87 (1)	2.08 (1)	2.947 (3)	170 (2)
N2—H2*A*⋯O4	0.86 (1)	2.13 (2)	2.868 (3)	144 (3)
O10—H10*A*⋯O3	0.82 (1)	2.09 (2)	2.832 (3)	150 (3)
O10—H10*A*⋯O4	0.82 (1)	2.24 (2)	2.743 (3)	120 (2)
O10—H10*B*⋯O3^ii^	0.83 (1)	2.05 (1)	2.869 (3)	171 (3)
O10—H10*B*⋯O9^ii^	0.83 (1)	2.43 (3)	2.898 (3)	117 (3)
C11—H11⋯O8^iv^	0.93	2.51	3.433 (3)	172

Symmetry codes: (i) 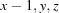; (ii) 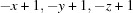; (iii) 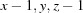; (iv) 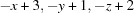.

**Fig. 1.**
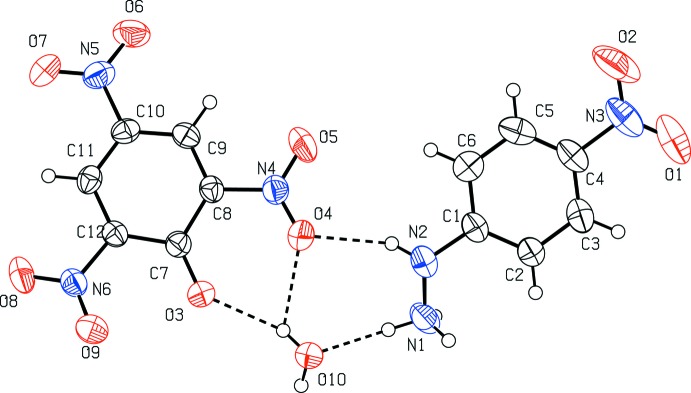
Molecular structure of (I) showing displacement ellipsoids drawn at the 50% probability level. H-bonds are shown in dashed lines.

**Fig. 2.**
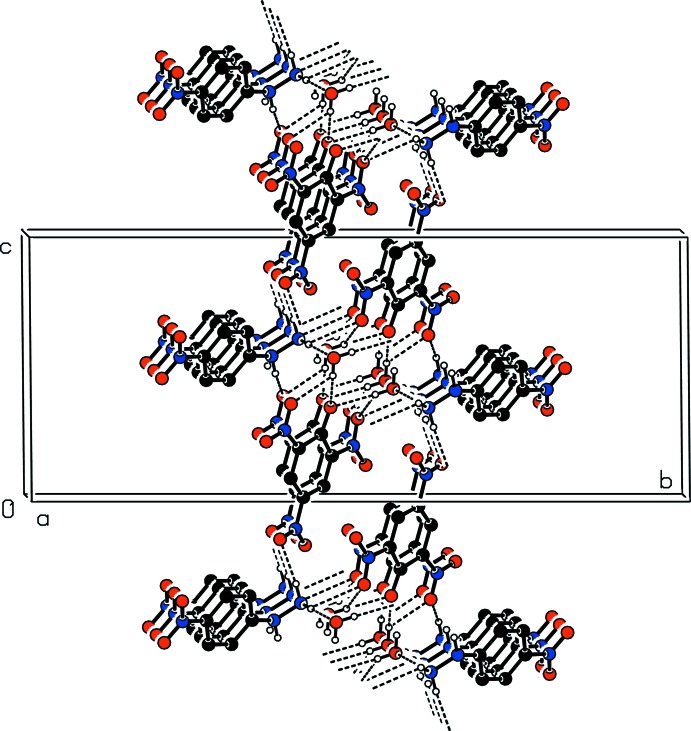
Part of the crystal structure of (I) showing the formation of a two-dimensional network parallel to (010). Hydrogen bonds are shown as dashed lines. For the sake of clarity, H atoms not involved in the motif have been omitted.

## supplementary crystallographic information

### 1. Comment

Cocrystal strategy has been often used due to its application in the fields of drug chemistry, physical material chemistry and biological crystallography. Picric acid, as a strong organic acid, is very frequently adopted to facilitate the crystallization of some difficult-crystallized organic bases (Herbstein & Kaftory, 1976; Dubost *et al.*, 1981; Harrison *et al.*, 2007; Peng *et al.*, 2011; Zeng *et al.*, 2011; Dey *et al.*, 2011). In this report, we report the 1:1 cocrystallized complex of 4-nitrophenylhydrazine and picric acid in 95% methanol solution.

In the title compound (I), the asymmetric unit consists of a 4-nitrophenyldrazinium cation, a picrate anion and a solvent water molecule (Fig.1). During the preparation of (I) the picric acid proton has been transferred to the terminal hydrazine atom N1. In the picrate molecule, the phenolate (O3—C7) bond distance is 1.245 (3) Å shows an indication of delocalization between the precursor single C—O bond and the benzene ring with three electron-withdrawing nitro groups. As a result, the neighbouring C7—C8 and C7—C12 bond lengths of 1.459 (3) Å and 1.452 (3) Å are longer by *ca* 0.08 Å than the mean distance of the other four C—C bonds. The C8—C7—C12 angle is about 10° smaller than the mean value (121.4°) of the other five benzene inner angles. The three nitro groups N4/O4/O5, N5/O6/O7 and N6/O8/O9 are twisted by 1.6 (3)°, 7.8 (3)° and 12.1 (3)° from the picrate benzene ring plane, respectively.

In the crystal, N—H···O and O—H···O hydrogen bonds link the components of the structure into a two-dimensional network parallel to (010) (Fig. 2). In addition, there are pairs of weak inversion related C—H···O hydrogen bonds within the two-dimensional network.

### 2. Experimental

All the reagents and solvents were used as obtained without further purification. 1:1 molar amount of 4-nitrophenylhydrazine (0.2 mmol, 30.6 mg) and picric acid (0.2 mmol, 45.8 g) were dissolved in 95% methanol (20.0 ml). The mixture was stirred for half an hour at ambient temperature and then filtered. The resulting yellow solution was kept in air for one week. Yellow needles of (I) suitable for single-crystal X-ray diffraction analysis were grown by slow evaporation of the solution at the bottom of the vessel. The crystals were filtered and dried in air. Yield: 60.2 mg, 75% (based on picric acid or 4-nitrophenylhydrazine).

### 3. Refinement

H atoms bonded to C atoms were positioned geometrically with C–H = 0.93 Å (aromatic) and refined in a riding-model approximation with [*U*_iso_(H) = 1.2*U*_eq_(aromatic C)]. H atoms bonded to N and O atoms were found in difference Fourier maps and refined with the constraints of N—H = 0.86 (1)Å and O—H = 0.82 (1) Å. The N1-bonded H···H distances were constrained by using the SADI command in SHELXL (Sheldrick, 2008). The water O10-bonded H···H distance was constrained to be 1.35 (1) Å. The isotropic displacment parameters of of N-bonded and water O10-bonded hydrogen atoms were set 1.2 times and 1.5 times of their parent atoms, respectively.

### Crystal data

**Table:**

C_6_H_8_N_3_O_2_^+^·C_6_H_2_N_3_O_7_^−^·H_2_O	*F*(000) = 824
*M**_r_* = 400.28	*D*_x_ = 1.669 Mg m^−^^3^
Monoclinic, *P*2_1_/*c*	Mo *K*α radiation, λ = 0.71073 Å
Hall symbol: -P 2ybc	Cell parameters from 924 reflections
*a* = 4.8483 (3) Å	θ = 2.3–21.5°
*b* = 28.798 (2) Å	µ = 0.15 mm^−^^1^
*c* = 11.6352 (8) Å	*T* = 299 K
β = 101.360 (1)°	Needle, yellow
*V* = 1592.70 (18) Å^3^	0.20 × 0.08 × 0.04 mm
*Z* = 4	

### Data collection

**Table:**

Bruker SMART CCD area-detector diffractometer	3643 independent reflections
Radiation source: fine-focus sealed tube	2487 reflections with *I* > 2σ(*I*)
Graphite monochromator	*R*_int_ = 0.045
φ and ω scans	θ_max_ = 27.5°, θ_min_ = 1.9°
Absorption correction: multi-scan (*SADABS*; Sheldrick, 1997)	*h* = −6→6
*T*_min_ = 0.971, *T*_max_ = 0.994	*k* = −37→36
16632 measured reflections	*l* = −14→15

### Refinement

**Table:**

Refinement on *F*^2^	Primary atom site location: structure-invariant direct methods
Least-squares matrix: full	Secondary atom site location: difference Fourier map
*R*[*F*^2^ > 2σ(*F*^2^)] = 0.063	Hydrogen site location: inferred from neighbouring sites
*wR*(*F*^2^) = 0.150	H atoms treated by a mixture of independent and constrained refinement
*S* = 1.05	*w* = 1/[σ^2^(*F*_o_^2^) + (0.0602*P*)^2^ + 0.5955*P*] where *P* = (*F*_o_^2^ + 2*F*_c_^2^)/3
3643 reflections	(Δ/σ)_max_ = 0.001
271 parameters	Δρ_max_ = 0.31 e Å^−^^3^
10 restraints	Δρ_min_ = −0.31 e Å^−^^3^

### Special details

**Table:**

Geometry. All e.s.d.'s (except the e.s.d. in the dihedral angle between two l.s. planes) are estimated using the full covariance matrix. The cell e.s.d.'s are taken into account individually in the estimation of e.s.d.'s in distances, angles and torsion angles; correlations between e.s.d.'s in cell parameters are only used when they are defined by crystal symmetry. An approximate (isotropic) treatment of cell e.s.d.'s is used for estimating e.s.d.'s involving l.s. planes.
Refinement. Refinement of *F*^2^ against ALL reflections. The weighted *R*-factor *wR* and goodness of fit *S* are based on *F*^2^, conventional *R*-factors *R* are based on *F*, with *F* set to zero for negative *F*^2^. The threshold expression of *F*^2^ > σ(*F*^2^) is used only for calculating *R*-factors(gt) *etc*. and is not relevant to the choice of reflections for refinement. *R*-factors based on *F*^2^ are statistically about twice as large as those based on *F*, and *R*- factors based on ALL data will be even larger.

### Fractional atomic coordinates and isotropic or equivalent isotropic displacement parameters (Å^2^)

**Table:**

	*x*	*y*	*z*	*U*_iso_*/*U*_eq_	
C1	−0.3571 (5)	0.67096 (8)	0.4377 (2)	0.0427 (6)	
C2	−0.5710 (5)	0.67706 (9)	0.3408 (2)	0.0473 (6)	
H2	−0.5922	0.6562	0.2786	0.057*	
C3	−0.7524 (6)	0.71420 (9)	0.3368 (3)	0.0523 (7)	
H3	−0.8968	0.7186	0.2722	0.063*	
C4	−0.7169 (6)	0.74447 (8)	0.4294 (3)	0.0505 (7)	
C5	−0.5048 (7)	0.73915 (10)	0.5256 (3)	0.0603 (8)	
H5	−0.4836	0.7603	0.5870	0.072*	
C6	−0.3246 (6)	0.70235 (10)	0.5302 (3)	0.0555 (7)	
H6	−0.1806	0.6983	0.5952	0.067*	
C7	0.7836 (5)	0.56180 (8)	0.7388 (2)	0.0365 (5)	
C8	0.6275 (5)	0.59770 (7)	0.7872 (2)	0.0344 (5)	
C9	0.6776 (5)	0.61004 (7)	0.9025 (2)	0.0361 (5)	
H9	0.5682	0.6327	0.9287	0.043*	
C10	0.8913 (5)	0.58870 (8)	0.9798 (2)	0.0375 (5)	
C11	1.0603 (5)	0.55567 (8)	0.9429 (2)	0.0363 (5)	
H11	1.2063	0.5420	0.9960	0.044*	
C12	1.0107 (5)	0.54316 (7)	0.8270 (2)	0.0345 (5)	
N1	−0.2290 (5)	0.59509 (8)	0.3776 (2)	0.0543 (6)	
H1A	−0.377 (3)	0.5818 (9)	0.394 (2)	0.065*	
H1B	−0.078 (3)	0.5783 (8)	0.399 (2)	0.065*	
H1C	−0.250 (4)	0.6027 (10)	0.3037 (11)	0.065*	
N2	−0.1620 (5)	0.63550 (8)	0.4446 (2)	0.0579 (6)	
H2A	−0.069 (6)	0.6290 (11)	0.5132 (14)	0.070*	
N3	−0.9135 (7)	0.78313 (9)	0.4265 (3)	0.0711 (8)	
N4	0.3991 (4)	0.62195 (7)	0.71219 (19)	0.0419 (5)	
N5	0.9370 (5)	0.60035 (8)	1.10213 (19)	0.0481 (5)	
N6	1.2004 (4)	0.50850 (7)	0.79502 (19)	0.0403 (5)	
O1	−1.1132 (6)	0.78583 (8)	0.3443 (3)	0.0885 (8)	
O2	−0.8693 (7)	0.81070 (9)	0.5084 (3)	0.1111 (11)	
O3	0.7262 (4)	0.54803 (7)	0.63542 (15)	0.0569 (5)	
O4	0.3387 (6)	0.61246 (9)	0.6108 (2)	0.1019 (10)	
O5	0.2717 (6)	0.65136 (10)	0.7518 (2)	0.0989 (10)	
O6	0.7656 (5)	0.62597 (7)	1.13576 (17)	0.0648 (6)	
O7	1.1385 (5)	0.58370 (8)	1.16900 (17)	0.0676 (6)	
O8	1.3571 (5)	0.48802 (8)	0.87253 (19)	0.0694 (6)	
O9	1.2022 (4)	0.50150 (8)	0.69233 (18)	0.0697 (6)	
O10	0.2853 (4)	0.54747 (6)	0.43546 (16)	0.0545 (5)	
H10B	0.264 (7)	0.5203 (5)	0.413 (2)	0.082*	
H10A	0.362 (7)	0.5483 (10)	0.5049 (12)	0.082*	

### Atomic displacement parameters (Å^2^)

**Table:**

	*U*^11^	*U*^22^	*U*^33^	*U*^12^	*U*^13^	*U*^23^
C1	0.0376 (13)	0.0414 (13)	0.0506 (15)	0.0074 (10)	0.0123 (11)	0.0029 (11)
C2	0.0436 (14)	0.0457 (14)	0.0508 (15)	0.0078 (11)	0.0049 (12)	−0.0036 (12)
C3	0.0426 (15)	0.0480 (15)	0.0648 (18)	0.0083 (12)	0.0067 (13)	0.0059 (14)
C4	0.0498 (15)	0.0353 (13)	0.0716 (19)	0.0075 (11)	0.0248 (14)	0.0055 (13)
C5	0.074 (2)	0.0456 (15)	0.066 (2)	0.0037 (14)	0.0243 (17)	−0.0121 (14)
C6	0.0565 (17)	0.0579 (17)	0.0494 (16)	0.0044 (13)	0.0038 (13)	−0.0049 (13)
C7	0.0354 (12)	0.0358 (12)	0.0376 (13)	0.0002 (10)	0.0059 (10)	−0.0009 (10)
C8	0.0320 (11)	0.0294 (11)	0.0407 (13)	−0.0004 (9)	0.0041 (10)	0.0015 (9)
C9	0.0375 (12)	0.0301 (11)	0.0423 (14)	−0.0018 (9)	0.0120 (10)	−0.0013 (10)
C10	0.0440 (13)	0.0336 (12)	0.0346 (13)	−0.0063 (10)	0.0069 (10)	−0.0033 (9)
C11	0.0354 (12)	0.0349 (12)	0.0363 (13)	−0.0043 (10)	0.0011 (10)	0.0038 (10)
C12	0.0321 (12)	0.0303 (11)	0.0413 (13)	−0.0012 (9)	0.0081 (10)	−0.0009 (9)
N1	0.0623 (15)	0.0477 (13)	0.0567 (15)	0.0216 (12)	0.0210 (12)	0.0058 (11)
N2	0.0523 (14)	0.0538 (14)	0.0629 (16)	0.0191 (11)	−0.0006 (12)	−0.0046 (12)
N3	0.077 (2)	0.0415 (14)	0.105 (2)	0.0169 (13)	0.0438 (18)	0.0148 (15)
N4	0.0409 (11)	0.0384 (11)	0.0444 (13)	0.0028 (9)	0.0040 (9)	0.0026 (9)
N5	0.0614 (14)	0.0457 (12)	0.0366 (12)	−0.0035 (11)	0.0087 (11)	−0.0022 (10)
N6	0.0341 (10)	0.0359 (10)	0.0500 (13)	0.0014 (8)	0.0067 (9)	−0.0026 (9)
O1	0.0673 (15)	0.0633 (15)	0.140 (2)	0.0261 (12)	0.0334 (16)	0.0306 (15)
O2	0.149 (3)	0.0583 (15)	0.135 (3)	0.0392 (16)	0.052 (2)	−0.0139 (16)
O3	0.0600 (12)	0.0658 (12)	0.0397 (10)	0.0221 (10)	−0.0030 (9)	−0.0134 (9)
O4	0.128 (2)	0.0933 (18)	0.0613 (15)	0.0675 (16)	−0.0387 (14)	−0.0269 (13)
O5	0.0998 (19)	0.121 (2)	0.0703 (16)	0.0799 (17)	0.0031 (13)	−0.0105 (15)
O6	0.0896 (16)	0.0642 (13)	0.0453 (11)	0.0148 (11)	0.0250 (11)	−0.0069 (9)
O7	0.0718 (14)	0.0810 (15)	0.0410 (11)	0.0095 (11)	−0.0104 (10)	−0.0080 (10)
O8	0.0686 (13)	0.0686 (13)	0.0667 (14)	0.0360 (11)	0.0026 (11)	0.0090 (11)
O9	0.0672 (13)	0.0888 (16)	0.0532 (13)	0.0298 (12)	0.0125 (10)	−0.0155 (11)
O10	0.0654 (13)	0.0531 (11)	0.0411 (11)	0.0203 (10)	0.0008 (9)	−0.0079 (9)

### Geometric parameters (Å, º)

**Table:**

C1—N2	1.384 (3)	C10—N5	1.437 (3)
C1—C2	1.385 (4)	C11—C12	1.371 (3)
C1—C6	1.391 (4)	C11—H11	0.9300
C2—C3	1.380 (4)	C12—N6	1.454 (3)
C2—H2	0.9300	N1—N2	1.403 (3)
C3—C4	1.370 (4)	N1—H1A	0.866 (10)
C3—H3	0.9300	N1—H1B	0.873 (10)
C4—C5	1.371 (4)	N1—H1C	0.874 (10)
C4—N3	1.462 (3)	N2—H2A	0.857 (10)
C5—C6	1.367 (4)	N3—O1	1.222 (4)
C5—H5	0.9300	N3—O2	1.227 (4)
C6—H6	0.9300	N4—O4	1.190 (3)
C7—O3	1.245 (3)	N4—O5	1.193 (3)
C7—C12	1.452 (3)	N5—O7	1.221 (3)
C7—C8	1.459 (3)	N5—O6	1.231 (3)
C8—C9	1.362 (3)	N6—O8	1.212 (3)
C8—N4	1.448 (3)	N6—O9	1.213 (3)
C9—C10	1.376 (3)	O10—H10B	0.825 (10)
C9—H9	0.9300	O10—H10A	0.820 (10)
C10—C11	1.378 (3)		
			
N2—C1—C2	122.3 (2)	C12—C11—H11	120.4
N2—C1—C6	117.6 (2)	C10—C11—H11	120.4
C2—C1—C6	120.0 (2)	C11—C12—C7	124.0 (2)
C3—C2—C1	119.7 (3)	C11—C12—N6	115.8 (2)
C3—C2—H2	120.1	C7—C12—N6	120.2 (2)
C1—C2—H2	120.1	N2—N1—H1A	111 (2)
C4—C3—C2	119.1 (3)	N2—N1—H1B	102 (2)
C4—C3—H3	120.4	H1A—N1—H1B	112.3 (15)
C2—C3—H3	120.4	N2—N1—H1C	108 (2)
C3—C4—C5	121.9 (2)	H1A—N1—H1C	112.3 (15)
C3—C4—N3	119.1 (3)	H1B—N1—H1C	110.8 (15)
C5—C4—N3	119.0 (3)	C1—N2—N1	119.9 (2)
C6—C5—C4	119.3 (3)	C1—N2—H2A	116 (2)
C6—C5—H5	120.3	N1—N2—H2A	111 (2)
C4—C5—H5	120.3	O1—N3—O2	123.9 (3)
C5—C6—C1	119.9 (3)	O1—N3—C4	118.6 (3)
C5—C6—H6	120.0	O2—N3—C4	117.5 (3)
C1—C6—H6	120.0	O4—N4—O5	120.0 (2)
O3—C7—C12	124.0 (2)	O4—N4—C8	119.8 (2)
O3—C7—C8	124.4 (2)	O5—N4—C8	120.2 (2)
C12—C7—C8	111.6 (2)	O7—N5—O6	122.6 (2)
C9—C8—N4	115.8 (2)	O7—N5—C10	119.2 (2)
C9—C8—C7	124.1 (2)	O6—N5—C10	118.2 (2)
N4—C8—C7	120.2 (2)	O8—N6—O9	121.8 (2)
C8—C9—C10	119.5 (2)	O8—N6—C12	118.6 (2)
C8—C9—H9	120.3	O9—N6—C12	119.6 (2)
C10—C9—H9	120.3	C7—O3—H10A	134.2 (9)
C9—C10—C11	121.5 (2)	H1B—O10—H10B	108 (3)
C9—C10—N5	119.5 (2)	H1B—O10—H10A	114 (2)
C11—C10—N5	119.0 (2)	H10B—O10—H10A	110.2 (17)
C12—C11—C10	119.2 (2)		
			
N2—C1—C2—C3	−177.3 (3)	C8—C7—C12—C11	3.9 (3)
C6—C1—C2—C3	−0.4 (4)	O3—C7—C12—N6	4.4 (4)
C1—C2—C3—C4	0.1 (4)	C8—C7—C12—N6	−177.02 (19)
C2—C3—C4—C5	0.5 (4)	C2—C1—N2—N1	−24.1 (4)
C2—C3—C4—N3	−178.4 (2)	C6—C1—N2—N1	159.0 (3)
C3—C4—C5—C6	−0.7 (4)	C3—C4—N3—O1	3.9 (4)
N3—C4—C5—C6	178.3 (3)	C5—C4—N3—O1	−175.0 (3)
C4—C5—C6—C1	0.3 (4)	C3—C4—N3—O2	−177.1 (3)
N2—C1—C6—C5	177.3 (3)	C5—C4—N3—O2	3.9 (4)
C2—C1—C6—C5	0.3 (4)	C9—C8—N4—O4	−178.5 (3)
O3—C7—C8—C9	174.6 (2)	C7—C8—N4—O4	0.1 (4)
C12—C7—C8—C9	−3.9 (3)	C9—C8—N4—O5	1.5 (4)
O3—C7—C8—N4	−3.9 (4)	C7—C8—N4—O5	−179.9 (3)
C12—C7—C8—N4	177.60 (19)	C9—C10—N5—O7	−175.1 (2)
N4—C8—C9—C10	−179.9 (2)	C11—C10—N5—O7	6.1 (3)
C7—C8—C9—C10	1.5 (3)	C9—C10—N5—O6	6.8 (3)
C8—C9—C10—C11	1.3 (3)	C11—C10—N5—O6	−172.1 (2)
C8—C9—C10—N5	−177.5 (2)	C11—C12—N6—O8	11.2 (3)
C9—C10—C11—C12	−1.3 (3)	C7—C12—N6—O8	−167.9 (2)
N5—C10—C11—C12	177.5 (2)	C11—C12—N6—O9	−167.3 (2)
C10—C11—C12—C7	−1.6 (3)	C7—C12—N6—O9	13.6 (3)
C10—C11—C12—N6	179.32 (19)	C12—C7—O3—H10A	155.2 (11)
O3—C7—C12—C11	−174.6 (2)	C8—C7—O3—H10A	−23.2 (12)

### Hydrogen-bond geometry (Å, º)

**Table:**

*D*—H···*A*	*D*—H	H···*A*	*D*···*A*	*D*—H···*A*
N1—H1*A*···O10^i^	0.87 (1)	2.05 (1)	2.916 (3)	177 (2)
N1—H1*B*···O10	0.87 (1)	1.94 (1)	2.809 (3)	172 (2)
N1—H1*B*···O9^ii^	0.87 (1)	2.56 (3)	2.908 (3)	105 (2)
N1—H1*C*···O6^iii^	0.87 (1)	2.08 (1)	2.947 (3)	170 (2)
N2—H2*A*···O4	0.86 (1)	2.13 (2)	2.868 (3)	144 (3)
O10—H10*A*···O3	0.82 (1)	2.09 (2)	2.832 (3)	150 (3)
O10—H10*A*···O4	0.82 (1)	2.24 (2)	2.743 (3)	120 (2)
O10—H10*B*···O3^ii^	0.83 (1)	2.05 (1)	2.869 (3)	171 (3)
O10—H10*B*···O9^ii^	0.83 (1)	2.43 (3)	2.898 (3)	117 (3)
C11—H11···O8^iv^	0.93	2.51	3.433 (3)	172

Symmetry codes: (i) *x*−1, *y*, *z*; (ii) −*x*+1, −*y*+1, −*z*+1; (iii) *x*−1, *y*, *z*−1; (iv) −*x*+3, −*y*+1, −*z*+2.

## References

[bb1] Bruker (2001). *SAINT-Plus* and *SMART* Bruker AXS Inc., Madison, Wisconsin, USA.

[bb2] Dey, S. K., Pramanik, A. & Das, G. (2011). *CrystEngComm*, **13**, 1664–1675.

[bb3] Dubost, J.-P., Léger, J.-M., Hickel, D. & Colleter, J.-C. (1981). *Acta Cryst.* B**37**, 751–754.

[bb4] Harrison, W. T. A., Swamy, M. T., Nagaraja, P., Yathirajan, H. S. & Narayana, B. (2007). *Acta Cryst.* E**63**, o3892.

[bb5] Herbstein, F. H. & Kaftory, M. (1976). *Acta Cryst.* B**32**, 387–396.

[bb6] Peng, Y., Zhang, A.-J., Dong, M. & Wang, Y.-W. (2011). *Chem. Commun.* **47**, 4505–4507. 10.1039/c1cc10400d 21387060

[bb7] Sheldrick, G. M. (1997). *SADABS* Bruker AXS Inc., Madison, Wisconsin, USA.

[bb8] Sheldrick, G. M. (2008). *Acta Cryst.* A**64**, 112–122. 10.1107/S0108767307043930 18156677

[bb9] Spek, A. L. (2009). *Acta Cryst.* D**65**, 148–155. 10.1107/S090744490804362X PMC2631630 19171970

[bb10] Zeng, B., Li, J. & Wang, G. (2011). *Acta Cryst.* E**67**, o1464. 10.1107/S1600536811018307 PMC3120377 21754835

